# Subacute combined degeneration induced by nitrous oxide inhalation: Two case reports

**DOI:** 10.1097/MD.0000000000019926

**Published:** 2020-05-01

**Authors:** Bing Zhao, Lixian Zhao, Zhixing Li, Renliang Zhao

**Affiliations:** a Department of Neurology, The Affiliated Hospital of Qingdao University, 16 Jiangsu Road, Qingdao, Shandong Province; b Shanghai Mental Health Center, Shanghai Jiaotong University School of Medicine, Shanghai Key Laboratory of Psychotic Disorders, Shanghai, China.

**Keywords:** proton Nitrous Oxide Subacute Combined Degeneration

## Abstract

**Rationale:**

Nitrous oxide (N_2_O), commonly known as “laughing gas,” is being increasingly abused by young people as a recreational drug; this can subsequently result in myelopathy and peripheral neuropathy, however, in China, few cases of neurologic deterioration by N_2_O abuse have been reported.

**Patient concerns:**

Herein, we present 2 patients who developed progressive limb weakness, numbness, and ataxia. Both of them had recreationally inhaled N_2_O intermittently for a long time.

**Diagnosis:**

Subacute combined degeneration (SCD) based on myelopathy and polyneuropathy after N_2_O abuse.

**Interventions:**

The 2 patients were treated with cessation of N_2_O inhalation, methylcobalamin capsule 500 μg tid (ter in die, which means 3 times a day), and compound vitamin B 1 tablet tid p.o.(per os, which means taken orally) for 1 month.

**Outcomes:**

The symptoms of altered sensation and the patients’ gait improved significantly.

**Lessons:**

The 2 cases raise awareness of the important mechanisms of N_2_O neurotoxicity, and clinicians should be made fully aware of such substance-related diseases. The incidence of N_2_O -induced neurotoxicity is insufficiently recognized and should be considered as an important cause of SCD, especially in adolescents with undifferentiated weakness and abnormal sensation; this is essential because serious complications such as irreversible paralysis can result from the absence of early diagnosis and treatment.

## 1 Introduction

N_2_O is a long-standing anesthetic and analgesic gas used commonly for surgical and dental procedures. Its recreational use, however, is rapidly increasing, especially among adolescents. When abused, N_2_O causes neurotoxicity by interfering with the bioavailability of vitamin B12; however, the criteria of N_2_O abuse is not yet known. Teresa et al^[1]^ have reported neurological and psychiatric complications, and even death, related to N_2_O abuse. According to the 2016 Global Drug Survey, 38.6% and 29.6% of N_2_O abuse occurred in the United Kingdom and the United States, respectively.^[2]^ However, the prevalence of N_2_O in China is still unknown. Only a few cases describing N_2_O -induced SCD in China have been reported yet. We herein present 2 cases of N_2_O abusers who manifested with myelopathy and polyneuropathy.

## 2 Case report

### 2.1 Case 1

A 21-year-old male presented with rapidly progressing numbness and weakness in the limbs, which initially began in the lower limbs 45 days before and subsequently spread to the upper limbs. He stated that he had difficulty in climbing stairs and squatting and was unable to walk. The patient had gained 25 kg weight but had no autonomic dysfunction or incontinence. His medical history was significant for N_2_O abuse, which was characterized by the inhalation of 16 to 24 L canister each time for 5 times a week during the previous month. The N_2_O was obtained from a nightclub and was inhaled through gas-filled balloons.

Our initial neurological examination revealed a normal mental status and cranial nerve function. Evaluation of muscle function and strength using the manual muscle test revealed that the upper limbs reduced to grade 4+, but that of lower limbs reduced to grade 3 to 4. Besides, the muscular tension decreased without muscle fasciculation. Additionally, the tendon reflex was absent bilaterally and the plantar response was negative on both sides. The bilateral heel-knee-shin test was unstable, whereas the bilateral finger-nose-finger test was stable. Furthermore, the patient exhibited a positive Romberg sign and equivocal Babinski sign. Sensory examination revealed that algesthesia and thermesthesia decreased symmetrically below the T2 level, whereas bilateral topesthesia, stereognosis, and vibration sensation were weakened. The patient walked with a wide-based and steppage gait.

Initial laboratory tests revealed elevated vitamin B12 (>1475 pmol/L, reference range 150–652.54 pmol/L) and homocysteine (48.47 μmol/L, reference range 4.0–15.4 μmol/L), normal red blood cells, hemoglobin, and mean corpuscular volume (MCV). Since the patient had taken mecobalamine tablets before the blood test, his vitamin B12 level had presumably been corrected. Serum copper, zinc, and ceruloplasmin levels and thyroid function were within normal ranges. He showed negative results for human immunodeficiency virus (HIV) antibody, serologic tests for syphilis (STS), Epstein-Barr virus, cytomegalovirus, and the autoimmune profile. Cerebrospinal fluid tests were normal with no evidence of albuminocytological dissociation.

**Figure 1:**
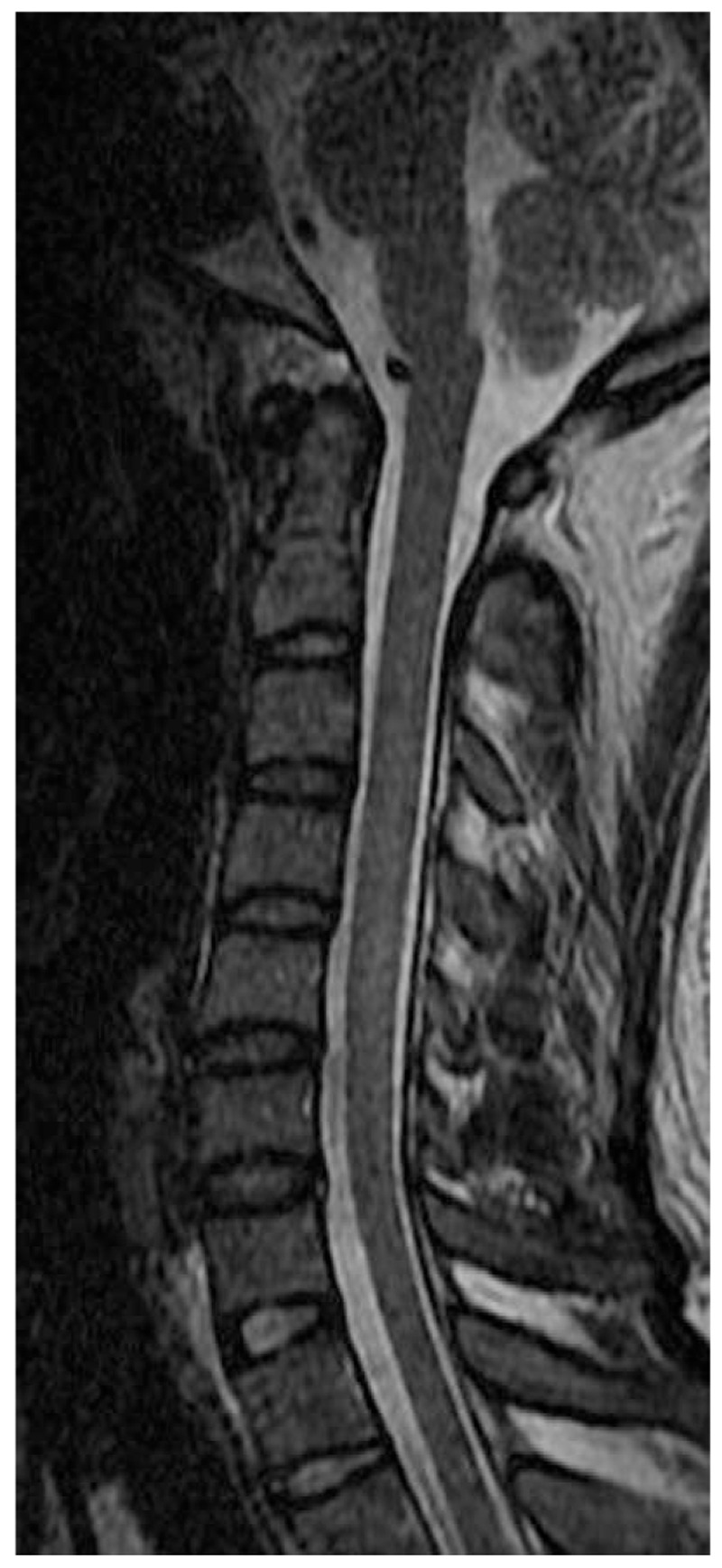
Sagittal magnetic resonance imaging demonstrates increased T2 signal in the posterior columns of the cervical spinal cord from C2 to C6.

**Figure 2:**
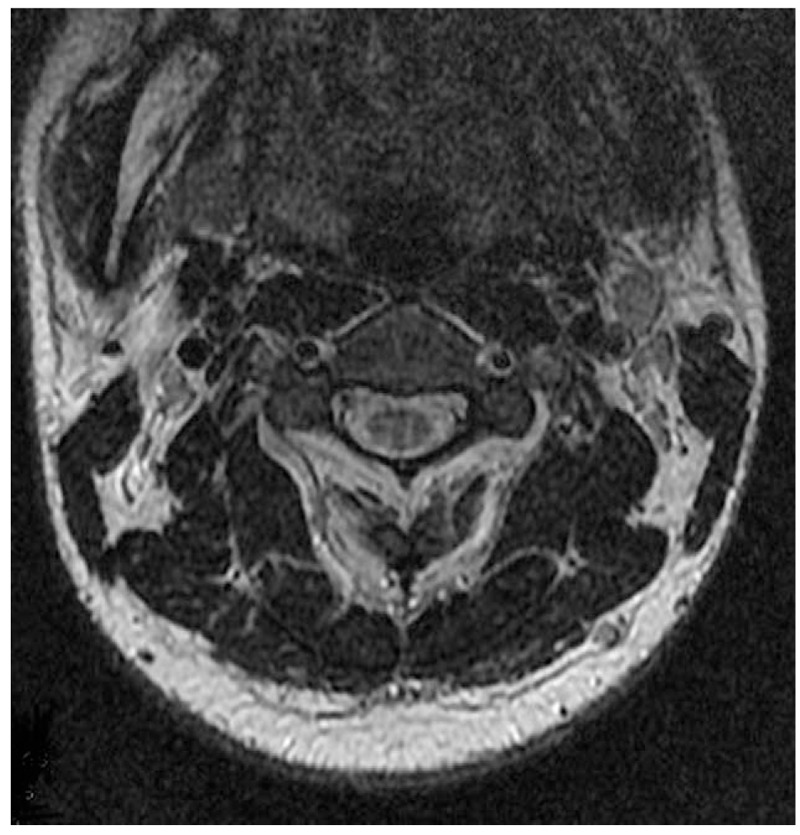
Axial magnetic resonance imaging, increased T2 signal is localized in the posterior columns with“rabbit ears sign”.

Magnetic resonance imaging (MRI) of the spinal cord demonstrated long segmental hyperintense lesion from C2 to C6 level in the posterior column (Figs. 1 and 2), whereas MRI of the brain showed unremarkable findings. Electromyography revealed sensorimotor polyneuropathy, which was dominated by demyelination.

Based on the abuse history, clinical manifestations, and auxillary examination, the patient was diagnosed with N_2_O -induced SCD. We started treatment with cessation of N_2_O inhalation, methylcobalamin capsule (500 μg tid p.o.), compound vitamin B (1 tablet tid p.o.), and rehabilitation therapy for a duration of 23 days. The symptoms of numbness improved, but the patient still had difficulty with walking independently. He was then transferred to a local hospital for subsequent rehabilitation therapy. Follow-up calls revealed that the patient could walk independently 4 months after discharge.

### 2.2 Case 2

An 18-year-old girl presented to the emergency department with progressive numbness and weakness in her upper and lower limbs for the past 15 days. The patient also reported a persistent “pins and needles” sensation in her lower limbs. She showed no autonomic dysfunction or incontinence; however, her weight had increased by 7.5 kg. Her medical history was significant for N_2_O abuse with an average of 40 L/day during the last month. She had bought the N_2_O (compressed in a 40-L cylinder) from her friend to relax at home, and she inhaled the gas through a mask with a pipe connected to the cylinder. The patient had received hyperbaric oxygen therapy a week previously, which was not available in our hospital (She did not state her detailed therapeutic regimen).

Our initial neurological examination revealed an anemic face with a normal mental status and cranial nerve function. Evaluation of muscle function and strength using the manual muscle test revealed that upper limbs reduced to grade 4+, whereas the proximal and distant lower limbs reduced to grade 4 and 0, respectively. Additionally, the muscular tension decreased without muscle fasciculation. Tendon reflex was absent bilaterally, and the plantar response was negative on both sides. The bilateral heel-knee-shin test was unstable; however, the bilateral finger-nose-finger test was stable. Sensory examination revealed that algesthesia and thermesthesia decreased symmetrically below the T4 level, whereas bilateral topesthesia, stereognosis, and vibration sensation were weakened.

Initial laboratory tests revealed a decreased level of red blood cell count (2.81 × 10^12^ cells/L, reference range 3.8–5.1 × 10^12^ cells/L) and hemoglobin (93 g/L, reference range 115–150 g/L), normal MCV (99.3 fL, reference range 82–100 fL), elevated homocysteine (48.47 μmol/L, reference range 4.0–15.4 μmol/L), and normal vitamin B12 (180 pmol/L, reference range 150–652.54 pmol/L). Serum copper, zinc, and ceruloplasmin levels and thyroid function were within normal ranges. The patient tested negative for HIV antibody, STS, Epstein-Barr virus, cytomegalovirus, and the autoimmune profile. The cerebrospinal fluid tests were normal with no evidence of albuminocytological dissociation.

**Figure 3:**
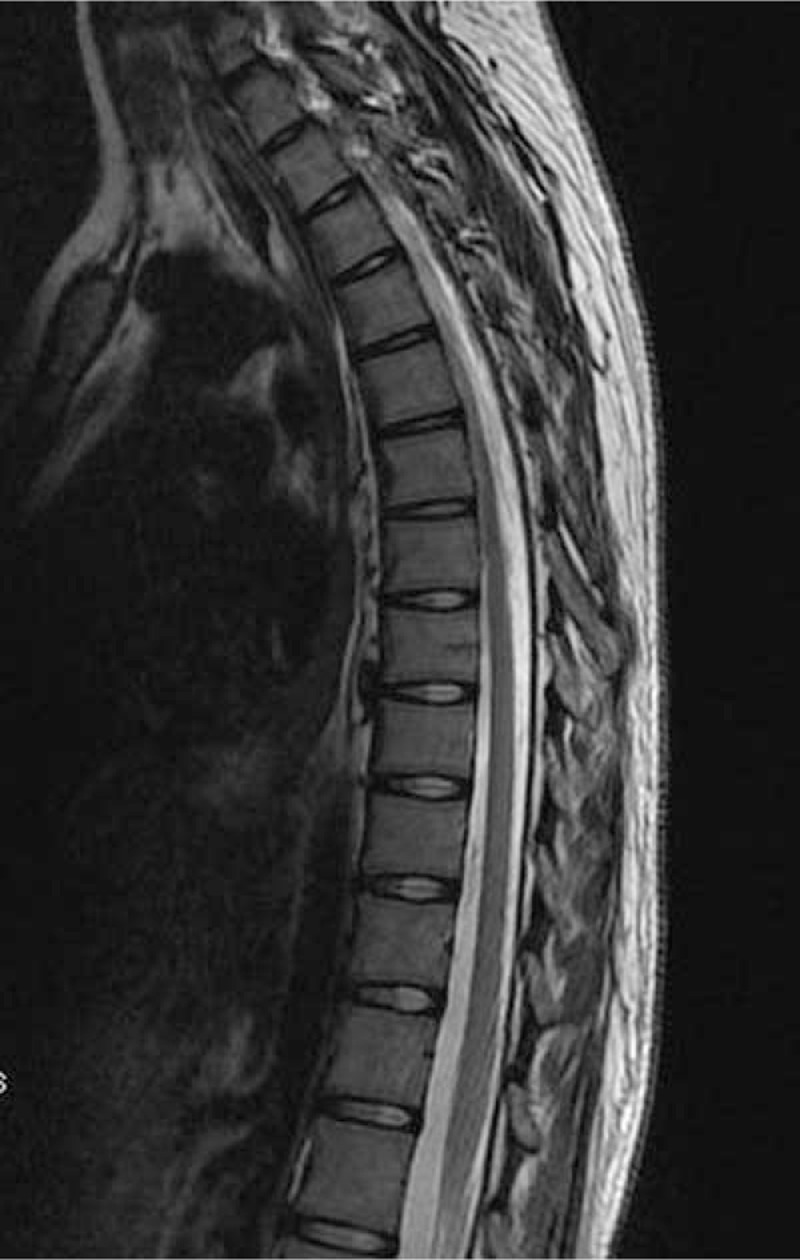
Sagittal imaging demonstrates increased T2 signal in the posterior columns of the thoracic spinal cord from T3 to T6.

**Figure 4:**
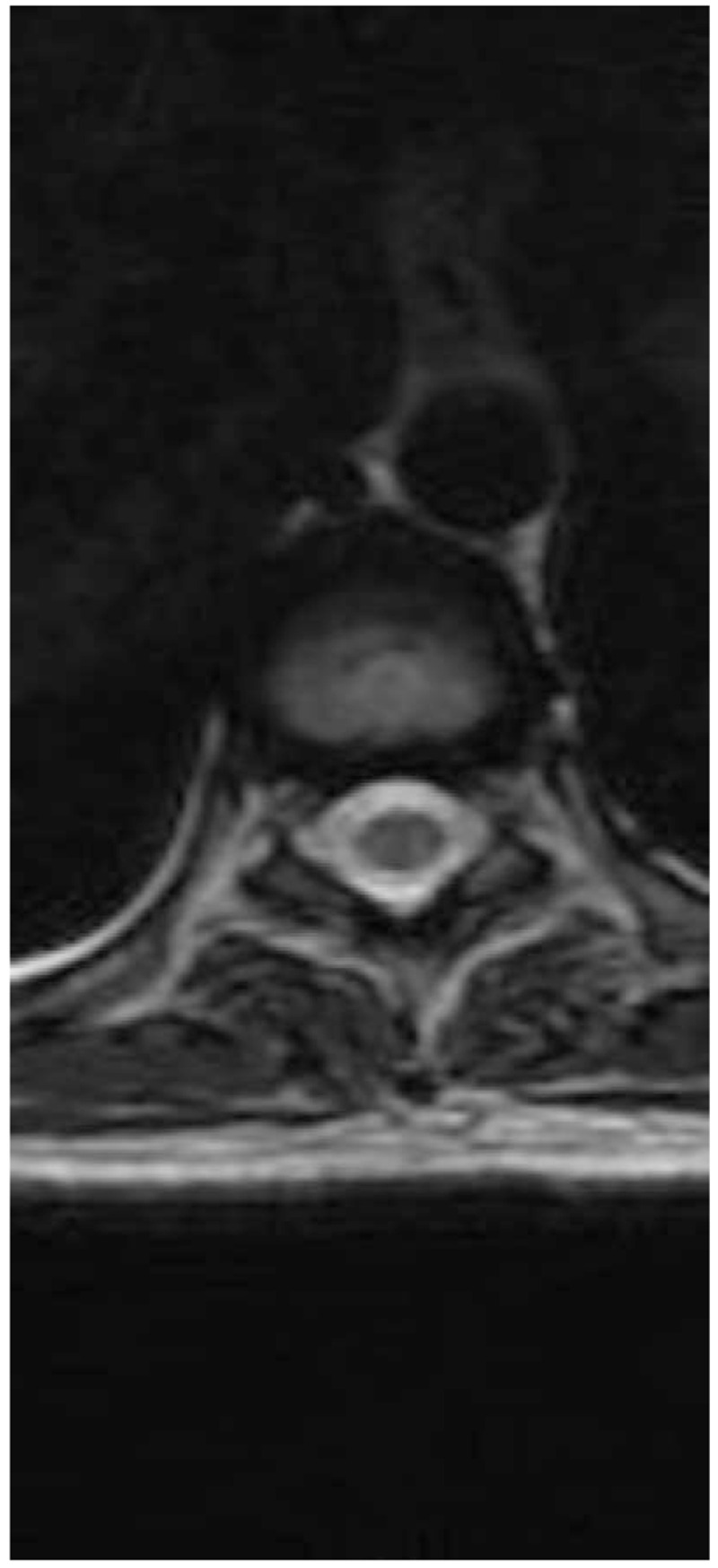
Axial sequences resonance imaging, increased T2 signal is localized in the posterior columns with “rabbit ears sign."

MRI of the spinal cord demonstrated segmental hyperintense lesion from level T3 to T6 in the posterior column (Figs. 3 and 4). MRI of the brain showed unremarkable findings. Electromyography revealed sensorimotor polyneuropathy, which was dominated by axonal injury and demyelination.

Based on the abuse history, clinical manifestation, and auxillary examination, the patient was diagnosed with N_2_O -induced SCD and anemia. She was treated with cessation of N_2_O inhalation and smoke, additional methylcobalamin capsule (500 ug tid p.o.), compound vitamin B (one tablet tid p.o.), and rehabilitation therapy for 1 month. The symptoms of sensation and gait improved significantly. The patient refused to repeat electromyography and MRI post-discharge. She was reviewed incidentally at 9 months after discharge and was noted to be free of any disabling neurological symptoms.

The patients have provided their consent to publish the case report, and the consent procedure was approved by the Ethics Committee of the Affiliated Hospital of Qingdao University.

## 3 Discussion

According to the Global Drug Survey 2016, N_2_O abuse is rapidly spreading around the world, and has become the seventh most popular recreational drug.^[3]^ Though potential complications are relatively rare, awareness is critical and can contribute to differential diagnosis when young patients develop unusual and otherwise unexplained symptoms. It is important to note that most N_2_O users underestimate the adverse effects and believe that it is safe to use since it is legal. An array of complications can arise from compressed N_2_O; the forced air can dissect through soft tissues producing complications like pneumomediastinum and pneumothorax.^[4]^ Additionally, frostbite injury from the canister, myocardial infarction, and multiple asphyxia-related deaths may be caused by N_2_O abuse.^[5]^ Moreover, N_2_O prevents the activation of cobalamin (that can result in vitamin B12 deficiency), which is being increasingly reported as SCD.^[6]^ In the spine, it can be characterized by dorsal column lesion with sensory disorders (paresthesia and proprioception disorder) and may progress to the lateral corticospinal tract accompanied by weakness and hyperreflexia.^[7]^ On MRI, it is characteristically identified by an abnormal T2 signal in the dorsal column, which is indicated by the “inverted V sign" or “rabbit ears sign.”^[8,9]^ Both the patients were suffering from SCD with weakness, paresthesia, and gait ataxia. Additionally, they also developed sensorimotor neuropathy with demyelinating and/or axonal injuries consistent with the pathophysiological mechanism of vitamin B12 deficiency.

Previous data have shown that in humans, 70% N_2_O causes methionine synthase activity to decline by 50% within 46 to 120 minutes and become almost completely inactive within 200 minutes.^[10]^ The harmful effects of excessive use of N_2_O are secondary because it interferes with the action of vitamin B12. N_2_O oxidizes cobalt ions in vitamin B12, thereby resulting in its inactivation. This leads to a reduction in the homocysteine-methionine cycle, which prevents methylation of myelin proteins, thereby leading to demyelination. A meta-analysis^[11]^ that was performed with 100 patients revealed that three-quarters of the patients with N_2_O -related toxicity showed a low vitamin B12 status (<150 pmol/L). However, studies have shown that the elevation of homocysteine and methylmalonic acid may occur earlier than vitamin B12 deficiency^[10,12]^ and is highly sensitive to the diagnosis of vitamin B12 deficiency. Both of our paients displayed elevated homocysteine; methylmalonic acid levels could, however, not be determined due to limitations in testing equipment. They had normal Vitamin B12 at the time of discharge; however, we did not monitor the level of vitamin B12 subsequently.

N_2_O can reduce anxiety and cause euphoria about 1 minute after inhalation, which can then completely subside after 2 minutes.^[13]^ It has been suggested that the risk of neurological impairment increases with N_2_O consumption of >80 g/day.^[14]^ Jan et al^[13]^ reported that repetitive use (50–100 bulbs) of N_2_O within 3 hours or heavy use over a prolonged time (ie, more than 10–20 bulbs everyday for 10 days) could cause deficiency of vitamin B12. Furthermore, clinical symptoms may also be related to the basal serum vitamin B12 levels. Waclawik et al^[10]^ reported that most of the patients with normal serum vitamin B12 require long-term and repeated exposure to N_2_O to damage the nerves, whereas patients with vitamin B12 deficiency will experience clinical symptoms even with small amounts of N_2_O.

Considering prognosis, it has been reported that SCD is significantly improved within 21 days after treatment. The speed of recovery may vary according to the degree of damage to the spinal cord and peripheral nerve.^[15]^

Regarding treatment, cessation of exposure and supplementation of vitamin B12 are essential treatments for N_2_O -induced SCD.^[16]^ Vitamin B12 therapy can be administered orally or intramuscularly. Our patients received 1500 μg/day according to the recommended dosage (1000–2000 μg/day)^[17]^ and additionally received rehabilitation training. Both recovered after approximately 1 month but refused reexamination with electromyography and MRI.

Our cases showed symmetric abnormal signal in the dorsal columns of the cervical and thoracic cord, which were characterized by a “rabbit ears sign." Besides, both the patients had myelopathy and polyneuropathy, which are necessary for clinicians to make a differential diagnosis. A limitation of our report is that the follow-ups were not integrated; the patients were reviewed through telephone, but they refused to be reexamined with electromyogram and MRI. The effectiveness of our treatment would be an important complement to the case literature, which can contribute to establish the potential neurologic damage associated with N_2_O -induced SCD and the course of recovery.

## 4 Conclusion

Abuse of N_2_O has been increasing rapidly in several countries. The lack of epidemiological studies of N_2_O abuse in China had concerned us. The criteria for N_2_O abuse, the risk factors for N_2_O, and the precise treatment options need to be explored further. It is essential that all frontline health care providers be aware of the risks associated with inhalant abuse to consult high-risk individuals before long-term harmful effects can occur. Familiarity with the symptoms of N_2_O neurotoxicity in situations of recreational exposure can also help to accelerate the identification and treatment of this debilitating disease.

## Author contributions

**Conceptualization:** Bing Zhao, Renliang Zhao.

**Investigation:** Bing Zhao.

**Resources:** Bing Zhao, Lixian Zhao.

**Software:** Bing Zhao, Lixian Zhao.

**Supervision:** Renliang Zhao.

**Validation:** Renliang Zhao.

**Visualization:** Zhixing Li.

**Writing – original draft:** Bing Zhao.

**Writing – review & editing:** Bing Zhao.
